# Partial Dopamine D2/3 Agonists and Dual Disorders: A Retrospective-Cohort Study in a Real-World Clinical Setting on Patients with Schizophrenia Spectrum Disorders and Cannabis Use Disorder

**DOI:** 10.2174/011570159X350599241214042724

**Published:** 2025-01-16

**Authors:** Giada Trovini, Ginevra Lombardozzi, Georgios D. Kotzalidis, Ilaria Pagano, Emanuela Amici, Valeria Giovanetti, Filippo Perrini, Andrea Fagiolini, Sergio De Filippis

**Affiliations:** 1 Clinic Villa Von Siebenthal, Rome, Italy;; 2 Department of Molecular and Developmental Medicine, Division of Psychiatry, University of Siena School of Medicine, Siena, Italy;; 3 NESMOS Department, Faculty of Medicine and Psychology, Sapienza University of Rome, Rome, Italy;; 4 Sant’ Andrea Hospital, School of Medicine and Psychology, Sapienza University of Rome, Rome, Italy;; 5 UOC DSMDP District ASL ROMA 6, TSMREE, Velletri, Rome, Italy

**Keywords:** Partial dopamine agonist antipsychotic drugs, aripiprazole, oral, aripiprazole, long-acting injectable, substance use disorders, cannabis use disorder, schizophrenia, schizoaffective disorder, comorbidity

## Abstract

**Introduction/Objective:**

Schizophrenia with substance use disorder is a complex clinical condition that may increase treatment resistance. Cannabis use disorder is frequently associated with psychosis and the causal link has still to be defined. Partial D_2/3_ agonists may ensure limbic dopamine release normalization while avoiding reduced frontocortical dopamine release, which would contribute to negative symptoms. We aimed to observe the clinical course of patients with schizophrenia comorbid with cannabis use disorder while being treated with oral or long-acting injectable D_2/3_ partial agonists.

**Methods:**

We observed 96 young adults with schizophrenia/schizoaffective disorder comorbid with cannabis use disorder during 18 months of treatment with aripiprazole long-acting injectable or oral aripiprazole or brexpiprazole. The assessment comprised Clinical Global Impressions-Severity, Positive And Negative Syndrome Scale, Brief Psychiatric Rating Scale, Barratt Impulsiveness Scale, and Visual Analog Scale for Craving.

**Results:**

Included were 17 women and 79 men (mean age = 26.89 ± 4.74 years). The sample responded favorably to treatment as assessed by all clinical scales, save for the impulsiveness scale which showed no significant change. The four treatment samples responded well without differences, but employing a general linear model, long-acting injectable aripiprazole and brexpiprazole were better and similar on all clinical and craving scales compared to oral aripiprazole and to other antipsychotics. Long-acting injectable aripiprazole fared better than brexpiprazole on general psychopathology, negative symptoms, and craving, while the reverse was true for global severity. However, the sample size imbalance did not allow for drawing strong conclusions. We found no significant treatment resistance in our 96-patient sample.

**Conclusion:**

Partial D_2/3_ agonists may treat comorbid schizophrenia/schizoaffective disorder and cannabis use disorder, improving the symptoms of both disorders and substance craving.

## 1 INTRODUCTION

Schizophrenia is a chronic illness that requires lifelong treatment. The primary goal in therapy is to treat the acute phase, prevent relapse, and guide the patient toward recovery and reintegration into the community. Approximately 50% of patients with schizophrenia may experience a significant clinical relapse within one year of drug discontinuing [1-3]. Careful selection of antipsychotics is an individualized risk-benefit decision based on both short- and long-term concerns, including the relative clinical efficacy, safety, and tolerability of antipsychotic medications [4]. Antipsychotics often need to be taken for very long periods of time, increasing the likelihood of adverse events that cause patients to discontinue medication and decrease adherence [5]. To overcome low adherence, clinicians may use long-acting injectable (LAI) formulations, which release the active drug during the 15 days to 6 months. There was hope that second-generation antipsychotics (SGAs) would be associated with lower relapse and rehospitalization rates compared to first-generation antipsychotics (FGAs), but this was not found to be the case [6].

Substance use disorders (SUDs) are common in patients with schizophrenia. A meta-analysis [7] found that the prevalence of any SUD in patients with schizophrenia or first-episode psychosis was 42%, with the most common SUDs being those related to illicit drugs (28%), cannabis (26%), alcohol (24%), and stimulants (7%). The treatment of patients with schizophrenia and a co-occurring SUD is particularly challenging. According to current guidelines, the best approach is to combine antipsychotic treatment with psychosocial interventions for addictive behaviors [8-10]. Currently, there is a lack of data on antipsychotics in adolescents with dual diagnosis [11], but SGAs are more acceptable than FGAs [12].

Although there are few advantages of one antipsychotic over another, when treating patients with co-occurring schizophrenia spectrum disorders and SUDs, SGAs may be preferable to FGAs because of their better tolerability and reduced tendency to induce extrapyramidal adverse effects. A comparison between the FGA LAI zuclopenthixol and the SGA LAI risperidone showed that the latter was superior in reducing both substance use and psychotic symptoms [13]. In addition, the potent dopaminergic blockade of FGAs may exacerbate craving [14].

Antipsychotics that are characterized by partial agonism of dopamine D_2_ receptors show a biphasic effect that includes functional antagonism in the mesolimbic dopamine pathway (where excessive dopamine activity is associated with positive symptoms) and functional agonist activity in the mesocortical pathway (where reduced dopamine activity is associated with negative symptoms and cognitive impairment). In addition, by avoiding complete blockade of the nigrostriatal or tuberoinfundibular pathways, these medications are associated with a low incidence of extrapyramidal symptoms and hyperprolactinemia [15].

Aripiprazole is the prototype of D_2_/D_3_ receptor partial agonists used in the treatment of schizophrenia and bipolar disorder [16, 17]. A large body of evidence indicates that is as effective as haloperidol and FGAs in the treatment of positive symptoms, more effective in the treatment of negative symptoms of schizophrenia, and causes fewer adverse effects [18]. In addition, aripiprazole acts as a partial agonist at serotonin 5-HT_1A_ receptors and as a potent antagonist at 5-HT_2A_ and 5-HT_2B_ receptors [19, 20].

Brexpiprazole shares with aripiprazole the partial agonism activity at D_2_, D_3,_ and serotonin 5-HT_1A_ receptors with a lower intrinsic efficacy at D_2_ receptors with respect to aripiprazole. Brexpiprazole also acts as an antagonist of 5-HT_2A_, 5.HT_2B_, 5-HT_7_, α_1A_ and α_1D_ adrenergic receptors [19, 20]. 5-HT_7_ blockade by brexpiprazole is predictive of an effect of the drug in improving cognitive dysfunction [21], whereas α_1_ adrenergic receptor blockade underlies the FDA-approved clinical application of brexpiprazole in agitation associated with Alzheimer’s disease [22-25].

Aripiprazole, brexpiprazole, and cariprazine share the D_2_/D_3_ receptor partial agonist property and for this reason, the term “third-generation antipsychotics” has been advanced [26]. We believe that this sort of proliferation of terms that may well be expressed otherwise does harm to the scientific debate, focusing on form rather than substance. We will maintain the FGA/SGA distinction and pool all newer than clozapine drugs among the latter. The atypical *vs*. typical (neuroleptic) antipsychotics distinction also did not find a reasonable neurochemical basis. For this reason, in this paper, we will maintain the term D_2_/D_3_ receptor partial agonists.

Dopaminergic dysfunction is known to be involved in addiction [27]. Thus, partial agonists of D_2_ receptors may be beneficial in reducing the rewarding effects of illicit drugs and restrain substance craving and relapse after withdrawal [28].

These properties, together with their efficacy on schizophrenia symptoms, lower risk of extrapyramidal side effects compared to FGAs, and lower risk of metabolic side effects compared to most SGAs [29], make aripiprazole and brexpiprazole attractive alternatives for the treatment of schizophrenia with comorbid SUDs. Despite differences in the neurobiology of each substance use–associated psychosis (our sample consisted of people with at least cannabis use disorder, but also other substance use was possible), partial dopamine D_2/3_ agonists have been proposed as potentially useful treatments in psychoses comorbid with SUD [30]. Brexpiprazole, thanks to its broader clinical target [31], is one of the substances that can address other comorbidities which often coexist in patients with psychosis and SUD [32].

This investigation provides insights into treatment strategies for patients with psychotic symptoms associated with SUD. Our intention was to evaluate the efficacy of aripiprazole and brexpiprazole in reducing psychotic symptoms and substance craving. In this study, we focused on the impact of aripiprazole and brexpiprazole on clinical and psychopathological outcomes compared to other antipsychotics.

## 2 MATERIALS AND METHODS

We conducted a retrospective observational study of inpatients and outpatients with a diagnosis of schizophrenia hospitalized at the Villa Von Siebenthal Neuropsychiatric Hospital in Rome, Italy. Recruitment started in June 2021 and ended in December 2022. Patients aged 18-35 years were eligible if they had a diagnosis of DSM-5/DSM-5-TR schizophrenia or schizoaffective disorder and cannabis use disorder.

This sample of patients is part of a sample used in a previously published study [33]; of the initial sample of 323, we followed 96 patients for 18 months. All were initially inpatients who were discharged after one month and then followed up as outpatients. Included were patients with cannabis use disorder and schizophrenia or schizoaffective disorder. Exclusion criteria were the presence of a comorbid major psychiatric disorder other than schizophrenia or schizoaffective disorder; suicide risk as assessed by the Columbia-Suicide Severity Risk Scale (C-SSRS) [34], *i.e*., a score of 4 or 5 on the suicidal ideation items or any “yes” response to the suicidal behaviour items; comorbidity with major organic diseases (autoimmune or systemic connective tissue disease, treatment-resistant hypertension, type 1 diabetes, metabolic syndrome, major cardiovascular disease, or major neurological disorders like Alzheimer’s disease and other dementias, cerebrovascular diseases, such as stroke, multiple sclerosis, Parkinson's disease, brain tumours, traumatic head injury, and neurological disorders related to malnutrition); history of epilepsy, head injury, electroencephalographic (EEG) abnormalities, and neurodevelopmental disorders; intelligence quotient (IQ)<75, as assessed by the Wechsler Adult Intelligence Scale (WAIS) [35]; unwillingness to participate and inability to sign the informed consent for themselves or, in the case of inability, unwillingness/refusal of the legal guardian to sign. Of the original 323-patient sample, 272 did not have schizophrenia or schizoaffective disorder (116 bipolar disorder, [36] depressive disorders, 57 borderline personality disorder, 55 attention-deficit/hyperactivity disorder, 5 other personality disorders, 2 mild intellectual disability, 1 autism spectrum disorder), thus leaving 119 patients to analyze. Of these, not all had a follow-up of 18-months available. Only 96 patients had this, the other 23 had still to reach the 18-month interval. This was due to the fact that some cases were included in the original study [33] until December 2022, so their 18-month data were not available at the time of our statistical analyses.

Patients provided written informed consent before participating in any study procedure. Both the informed consent form and the experimental procedures were approved by the Ethics Committee of the Health Authority of Rome 2 (study 331-306-00387; 19-November-2019), in accordance with internationally accepted criteria for ethical research. The study was conducted in accordance with the human rights principles adopted by the World Medical Association at the 18^th^ WMA General Assembly, Helsinki, Finland, June 1964, and subsequently amended at the 64^th^ WMA General Assembly, Fortaleza, Ceará, Brazil, October 2013.

We collected sociodemographic data on an appropriate datasheet. We assessed the psychopathology of the 96 follow-up patients using the Clinical Global Impressions-Severity Scale (CGI-S) [36], the Positive And Negative Syndrome Scale (PANSS) [37, 38], and the 24-item Brief Psychiatric Rating Scale (BPRS) (original version [39], Italian version, [40]) at baseline, 1 month, and 3, 6, 12, and 18 months. The Visual Analog Scale for Craving (VAScrav) [41] was used to assess craving. The latter rates craving from 0 (no craving) to 10 (the most intense craving according to the patient's experience). Impulsivity was assessed using the Barratt Impulsiveness Scale (BIS-11) [42]. We used clinical face-to-face or telephone interviews for all recruited patients. DSM-5 psychiatric diagnoses were made using the Structured Clinical Interview for DSM-5-Clinician Version (SCID-5-CV) [43]. Patients were regularly screened for drug use at enrolment and throughout the study.

Eleven patients were on oral aripiprazole, 13 patients on brexpiprazole, and 38 on LAI aripiprazole. The other 34 patients took FGAs, *i.e.,* haloperidol, promazine, and amisulpride, or other SGAs, *i.e.,* clozapine, lurasidone, olanzapine, paliperidone, quetiapine, and risperidone.

We enrolled SUD patients who were receiving specific pharmacological treatment for their SUD, such as methadone, buprenorphine, and naltrexone, or benzodiazepines and gabapentinoids. All drugs used in this study adhered to clinical prescription guidelines as recommended by each product’s monograph. Aripiprazole was used as oral 10-30 mg/day according to patients’ need, and as LAI at 400 mg/month, while brexpiprazole was rapidly titrated up to 4 mg/day, remaining stable thereafter. Despite our study was real-world, multiple pharmacotherapies were not allowed; patients received one of the aforementioned antipsychotic treatments and were allowed to add only their specific drug treatment for their SUD. All patients, after they were discharged from the hospital, were followed up as outpatients by the same treating physicians who adhered to monotherapy treatment patterns.

Our primary objective was to evaluate the efficacy of antipsychotics characterized by partial agonism of dopamine D_2_ receptors, specifically aripiprazole (oral formulation and LAI) and oral brexpiprazole (there is no LAI for this drug) in a group of patients diagnosed with schizophrenia/schizoaffective disorder and cannabis use disorder (CUD), using CGI-S, PANSS, BPRS, BIS-11, and VAScrav scores.

### 2.1 Statistical Analysis

Frequency distributions and descriptive statistics were used to analyze the sample. After testing the sample for normality of distribution with the Shapiro-Wilk test [44], we proceeded with parametric tests.

First, preliminary analyses were performed to verify the internal consistency of the instruments used in the study by calculating Cronbach's *alpha*. Then, descriptive analyses were performed, which included the calculation of frequencies, means, and standard deviations (SDs) of sociodemographic and clinical variables, including the entire sample and its subdivisions (aripiprazole oral *vs*. LAI *vs*. brexpiprazole).

Where possible, differences between groups were tested using multivariate analysis of variance (MANCOVA), and the significance of the main effect was analyzed using Pillai's Trace index. The Bonferroni correction of α for multiple comparisons was applied in case of significance.

To evaluate the effect of partial agonist treatment on CGI-S, BPRS, BIS-11, and PANSS scores, we used a general linear model (GLM) with repeated measures to assess changes over time.

*Post hoc* comparisons were performed using Bonferroni's correction [45]. Contingency tables were used for nonparametric variables, and the significance of the main effect was analyzed by the chi-squared test. We used SPSS v.27 for Mac software to perform all statistical analyses (IBM Corporation, Armonk, New York, 2019). Significance was set at *p* < 0.05.

## 3 RESULTS

Demographic and clinical variables of the sample are shown in Table **1**. The sample consisted of 17 women and 79 men. The mean age was 26.89 (SD = 4.74) years. The age of onset of cannabis use was 15.75 years (SD = 2.36), earlier than the mean ages of onset of psychotic symptoms and first hospitalization, which were 18.68 (SD = 2.97) and 19.81 years (SD = 4.15), respectively.

We also examined the use of psychoactive substances other than cannabinoids (Table **2**). Most of our sample (72%) reported polysubstance use. In particular, 58.3% of patients also used cocaine and 33.3% used alcohol.

### 3.1 Efficacy of Antipsychotic Treatment Over Time

To investigate the effect of partial agonist treatment on the clinical variables studied at different time points, we used a general linear model (GLM) with repeated measures, comparing assessments at the time of hospitalization (baseline) with those at 18 months. The analysis showed a significant main effect for all variables, allowing comparisons within and between groups, corrected with the Bonferroni method for multiple comparisons.

Table **3** shows a general decrease in the scores of all psychometric scales in the whole sample from baseline to 18 months. Statistically significant changes between baseline and endpoint are shown for all the scales. Moreover, the effect attributable to the interaction between time and partial agonists is significant only for the BPRS (77.67 at baseline *vs*. 45.08 at endpoint), and the PANSS Positive subscale (24.73 at baseline *vs*. 13.42 at endpoint), although it went close to statistical significance for the CGI-S and the PANSS Total Score. BIS-11 and PANSS Negative and General Psychopathology scores did not change significantly from baseline to 18 months.

### 3.2 Efficacy of Different Antipsychotics *vs*. Partial Dopamine Receptor Agonists

First, we evaluated the efficacy of aripiprazole and brexpiprazole by comparing them with various FGAs and other SGAs. The assessment was based on the administration of the above psychometric scales at the beginning of hospitalization and after 18 months. We also compared the specific effect of each class (aripiprazole, brexpiprazole, and other antipsychotics) on different psychopathological dimensions using a general linear model (GLM) with repeated measures.

**Fig. (1):**
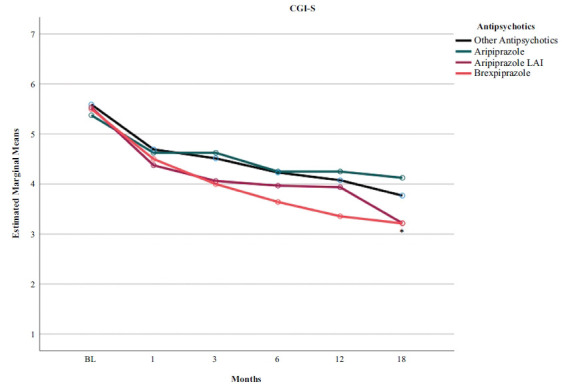
Time course of CGI-S scores according to the type of pharmacotherapy in a sample with comorbid schizophrenia and cannabis use disorder. BL, baseline. *p < 0.05 brexpiprazole *vs*. all others.

**Fig. (2):**
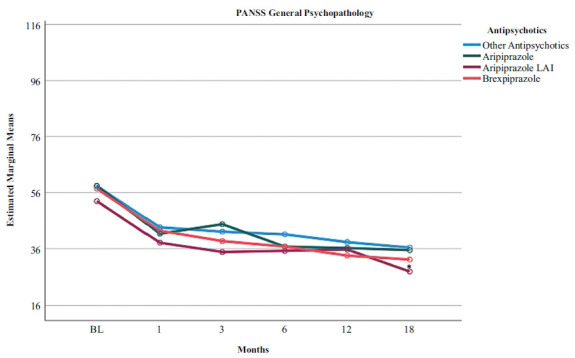
Time course of PANSS-General Psychopathology scores according to the type of pharmacotherapy in a sample with comorbid schizophrenia and cannabis use disorder. BL, baseline. *p < 0.05 LAI aripiprazole *vs*. all others.

**Fig. (3):**
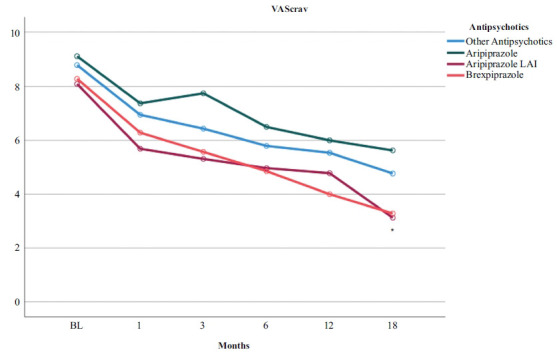
Time course of VAScrav scores according to the type of pharmacotherapy in a sample with comorbid schizophrenia and cannabis use disorder. BL, baseline. *p < 0.05 LAI aripiprazole *vs*. all others.

Table **4** shows a greater effect of brexpiprazole on CGI-S scores (Figs. **1** and **2**), whereas aripiprazole LAI significantly affected BPRS and VAScrav scores (Fig. **3**). Regarding the PANSS dimensions, patients on aripiprazole LAI scored 56.27 on the Negative subscale, which was statistically significant (*p* = 0.031) *vs*. other antipsychotics (25.00), aripiprazole (22.19), and brexpiprazole (22.04). Similarly, on the General Psychopathology subscale, patients on aripiprazole LAI scored better (20.42) *vs*. 47.37 for other antipsychotics, 47.99 for aripiprazole, and 44.79 for brexpiprazole (Fig. **2**). Scores on the PANSS positive symptoms subscale and the BIS-11 did not differ between the different drug regimens.

## 4 DISCUSSION

In this study, we evaluated 96 patients with schizophrenia/schizoaffective disorder and comorbid SUD. These patients were all treated with partial dopamine receptor agonists, including brexpiprazole, oral aripiprazole, aripiprazole LAI, and other antipsychotics. LAI aripiprazole improved BPRS scores and cravings better than all other treatments, while brexpiprazole improved CGI-S scores better than all others. We found no baseline-to-endpoint differences between brexpiprazole, oral aripiprazole, LAI aripiprazole, and other antipsychotics in terms of impulsiveness symptoms, as assessed by the BIS-11. We could conclude from our results that people with schizophrenia/schizoaffective disorder who have comorbid SUD do not respond less to brexpiprazole or aripiprazole treatment than patients treated with other antipsychotics, but may instead respond better. However, the limited statistical power of our sample and the relative differences between the four groups in sample size, despite sample homogeneity, do not allow us to draw firm conclusions.

Another issue worth to be considered is the efficacy of treatment with aripiprazole and brexpiprazole in reducing psychotic symptoms, and substance craving, and their ability to improve the global clinical status.

In this study, brexpiprazole appeared to be significantly more efficacious than other treatments on global symptom status measured by the CGI-S. Brexpiprazole may be better suited for the long-term treatment of adult schizophrenia because it has a favorable safety and tolerability profile, in addition to reducing both positive and negative symptoms, thereby achieving the goals of increasing patient socialization and community reintegration [46]. Previous studies of brexpiprazole support our findings. Correll *et al.* [47] carried out a multicentre, randomized, double-blind, placebo-controlled trial to assess the efficacy, safety, and tolerability of oral brexpiprazole 2 and 4 mg/day; they showed superior efficacy and good tolerability compared with placebo in patients with acute exacerbations of schizophrenia. In a recent study, Lombardozzi *et al.* [48] evaluated treatment response to brexpiprazole in patients with schizophrenia comorbid or not with SUD and substance craving in the SUD subsample. Brexpiprazole was associated with similar significant improvements from baseline in both groups at the 6-month endpoint. In addition, the comorbid and non-comorbid subsamples did not differ in treatment response, suggesting that SUD comorbidity did not inhibit or reduce antipsychotic response to the drug.

In this study, we found the superiority of LAI aripiprazole over other treatments on the 24-item BPRS, PANSS Negative and PANSS General Psychopathology scales, and VAScrav frequency and intensity. In our clinical experience, LAIs have several advantages over oral antipsychotics, including ensuring clinician awareness of nonadherence, reducing pill burden, and reducing the consequences of planned or unplanned treatment gaps [49]. Psychotic relapse is extremely distressing for patients and their families and is associated with multiple downstream effects on disease progression and brain structure, such as progressive reductions in grey and white matter structure and volume [50]. Relapses can also lead to reduced response to previously effective antipsychotics, potentially contributing to treatment resistance [51]. Co-occurring SUDs in people with schizophrenia add to the complexity of psychiatric comorbidity, which is associated with increased symptom severity, poorer course of illness, high rates of treatment nonadherence, and increased rates of relapse. In people with addiction, reduced dopamine function results in decreased sensitivity to nondrug stimuli and reduced inhibitory function of the frontal cortex [52]. These findings suggest that aripiprazole-induced dopamine release may be associated with increased activation of the anterior cingulate, which may control craving for alcohol and other substances [17]. Regarding the serotonergic system, the 5-HT_1A_ partial agonist effect of aripiprazole may modulate the prefrontal cortex to improve impulse control through projections from the raphe nucleus to the ventral tegmental area and nucleus accumbens [53]. Craving, despite the differences in the substance involved, that may extend from cocaine [54-56] and dopaminomimetics [57-59] to cannabinoids [60, 61], to nicotine [62], alcohol [63], and food [64, 65], may share common mechanisms involving dopamine receptors, probably countered by partial dopamine D_2_/D_3_ agonists [66].

In this investigation, LAI aripiprazole shows significant efficacy in reducing craving, and our data are clearly supported by various studies on the treatment of schizophrenia comorbid with SUD. The results of this study are consistent with previous literature. Aripiprazole produced significant improvements in psychotic symptoms and substance-related clinical outcomes, demonstrating efficacy in reducing craving and preventing relapse in patients with SUD [67]. LAI aripiprazole improved adherence and functioning in two patients with psychotic disorders and SUD [68], suggesting that it may be an effective and safe treatment option for dual disorders.

Once-monthly aripiprazole LAI improved craving and QoL at 1-year follow-up compared with paliperidone palmitate in initially hospitalized patients with psychosis comorbid with SUD [66]. Szerman *et al.* [69] carried out a multicentre, observational, retrospective study to evaluate the efficacy and impact of aripiprazole LAI in patients with schizophrenia with comorbid SUD. Severity was assessed using the Clinical Global Impressions Severity Scale for schizophrenia, and daily functioning and disability were assessed using the World Health Organization Disability Assessment Scale. At the one-year follow-up, aripiprazole improved both clinical status and quality of life, and reduced drug use, craving, and dependence severity.

In our study, the improvement in psychiatric symptoms is clinically meaningful and suggests that treatment with aripiprazole and brexpiprazole produces clinically relevant improvements in psychopathology. These findings suggest that both drugs may be effective in alleviating withdrawal symptoms associated with dopamine deficiency and, because of their broad receptor activity, may represent a new strategy for achieving balanced dopamine levels in the treatment of drug dependence. The co-occurrence of SUDs and psychosis is increasingly common and has been shown to worsen the longitudinal course of illness, reduce medication adherence and increase relapse rates. The treatment of schizophrenia is challenging, with the primary goal being to treat the acute phase, prevent relapse, and guide the patient to recovery. There are so many antipsychotics available today that it has become a challenge for psychiatrists to choose among them for each patient.

Long-term treatment may improve psychopathology, increase relapse prevention, reduce rehospitalizations, and improve outcomes in individuals with psychotic symptoms and comorbid substance use. Future studies will determine whether there are differences between the marketed partial D_2_/D_3_ agonists aripiprazole and brexpiprazole in the treatment of comorbid major psychiatric disorders and SUDs. For the moment, we may produce the following recommendations for clinicians and provide prompts for future research to clinical investigators. When dealing with people with comorbid SUDs and psychotic disorders like schizophrenia or schizoaffective disorder, try to persuade the patient to take an LAI like aripiprazole once monthly, which performed best in our study according to a general linear model; then try oral brexpiprazole that performed slightly better than oral aripiprazole according to the same model. The least recommendable treatments seem to be first-generation antipsychotics and other than D_2/3_ partial antagonist second-generation antipsychotics. Future research perspectives may consist in extending the observation period to two years. The other D_2/3_ partial antagonist we did not use here, cariprazine, could be a suitable comparator in future studies. Dealing with individual substances other than cannabinoids could provide clues as to neurobiological differences between various psychotic subtypes.

Another issue to deal with in future studies is how thought disorder responds to specific pharmacotherapy, in particular to those drugs we used in this study. Residual thought disorder is a neglected issue in current literature [70], and the scales used here only partially address it. The development of scales is welcome which could focus on the cognitive aspects of thought disorder and not only based on its positive and negative dimensions.

## 5 LIMITATIONS

Our study has several limitations. Our sample size was small and needs to be increased in order to draw stronger conclusions. Furthermore, the three dopamine partial agonist groups were not well-balanced, with the LAI group more than doubling the oral groups. In addition to the retrospective and uncontrolled design, another limitation is the allowed concomitant use of different psychotropic medications. In this study, we do not report on safety, which was assessed and will be the subject of a future study. Further, we did not measure plasma levels of aripiprazole or brexpiprazole in our patients nor did we perform genetic investigations regarding the metabolizer status of each patient to each drug received. The latter is not easy to obtain and may create problems with privacy and consent obtainment. While best therapeutic and safety windows have been identified for aripiprazole and its major active metabolite [71, 72], no such windows have been identified for brexpiprazole. It would be interesting to correlate in future studies the serum/plasma levels of brexpiprazole with clinical response. Despite its important limitations, our study suggests that drugs with partial agonist activity at dopamine D_2/3_ and serotonin 5-HT_1A_ receptors and LAI formulations maintain their antipsychotic efficacy in patients with schizophrenia/schizoaffective disorder and coexisting SUD. Our data should await replication in larger samples.

## CONCLUSION

The coexistence of cannabis use disorder and schizophrenia/schizoaffective disorder does not reduce the clinical response to antipsychotics endowed with partial D_2/3_ agonist properties. The treatment with such agents is associated with a strong reduction of craving symptoms. All antipsychotic treatment regimens were associated with significant improvements on all tested scales. Differences among D_2/3_ partial agonists oral and LAI and other SGA and FGA formulations were found only with the repeated-measures general linear model analysis and regarded oral brexpiprazole *vs*. others on global impressions, aripiprazole LAI *vs*. others on psychiatric symptom rating, general psychopathology, and negative symptomatology.

## Figures and Tables

**Table 1:** Demographic and clinical variables of the sample (17 women [17.7%] and 79 men [82.3%]).

**Variable**	**Mean**	**SD**
Age	26.89	4.74
Age at onset of psychosis	18.68	2.97
Age at first hospitalization	19.81	4.15
Age at onset of cannabis use	15.75	2.36
Time since beginning of cannabis use and onset of psychosis	2.92	2.61
Relapses	0.84	1.18

**Abbreviation**: SD, standard deviation.

**Table 2:** Substance use in the entire sample*.

-	**N**	**%**
Polysubstance use	70	72.2%
**Type of Substance**		
Cocaine	56	58.3%
Opioids	8	8.3%
Amphetamines	24	25%
Hallucinogens	14	14.6%
Alcohol	32	33.3%
Benzodiazepines	4	4.2%

**Note**: *The total number of substance use patterns exceeds the total number of patients because multiple substance use patterns may exist for the same patient.

**Table 3:** Repeated-measures General Linear Model (GLM), estimated marginal means, baseline *vs*. 18-month endpoint in whole sample.

-			**Time**		**Time x Partial Agonists**	
-	Baseline*	18 Months*	***F***	***p***	***F***	***p***
CGI-S	5.54	3.53	**468.14**	**<.001**	3.24	0.075
BIS-11 Total score	90.56	73.61	**166.33**	**<.001**	0.05	0.829
BPRS	77.67	45.08	**66.18**	**<.001**	**5.21**	**0.025**
PANSS Total score	109.85	63.10	**86.92**	**<.001**	3.43	0.067
PANSS Negative scale	28.94	16.83	**165.79**	**<.001**	2.28	0.134
PANSS Positive Scale	24.73	13.42	**67.66**	**<.001**	**4.61**	**0.034**
PANSS General Psychopathology	56.35	32.75	**119.82**	**<.001**	1.83	0.179

**Abbreviations**: BIS-11, Barratt Impulsiveness Scale; BPRS, 24-item Brief Psychiatric Rating Scale; CGI-S, Clinical Global Impressions scale, Severity; PANSS, Positive And Negative Syndrome Scale. **Note: ** Significance in bold. *Estimated marginal means, representing the average predicted outcomes of the model for each level of the independent variables, taking into account the effects of time and partial agonists in the model.

**Table 4:** Repeated measures general linear model (GLM).

**-**	**OAPs**	**Aripiprazole**	**Aripiprazole LAI**	**Brexpiprazole**	**F**	***p***
CGI-S	4.68	4.75	4.38	**4.36**	**4.87**	**0.003**
BIS-11 Total	82.26	83.25	81.36	82.61	0.10	0.961
BPRS	63.77	70.71	**56.27**	61.71	**3.10**	**0.031**
PANSS Total	91.45	91.00	79.70	85.50	2.60	0.057
PANSS Negative	25.00	22.19	**20.84**	22.04	**3.10**	**0.031**
PANSS Positive	19.26	21.81	18.42	18.50	0.715	0.546
PANSS General Psychopathology	47.36	47.00	**40.42**	44.79	**3.53**	**0.018**
VAScrav frequency	5.83	6.44	**4.58**	5.29	**3.89**	**0.012**
VAScrav intensity	6.78	7.38	**5.61**	5.79	**4.87**	**0.004**

**Abbreviations**: BIS-11, Barratt Impulsiveness Scale; BPRS, 24-item Brief Psychiatric Rating Scale; CGI-S, Clinical Global Impressions scale, Severity; OAPs, other antipsychotics; PANSS, Positive And Negative Syndrome Scale; VAScrav, visual analogue scale for craving. **Note: ** Significance in bold.

## Data Availability

The raw data supporting the conclusions of this article will be provided by the corresponding author upon reasonable request without reservation.

## LIST OF ABBREVIATIONS

EEG: Electroencephalographic
GLM: General Linear Model
LAI: Long-acting Injectable
SDs: Standard Deviations
SGAs: Second-generation Antipsychotics
SUDs: Substance Use Disorders
